# Ionic Liquids Chemical Stress Triggers Sphingoid Base Accumulation in *Aspergillus nidulans*

**DOI:** 10.3389/fmicb.2019.00864

**Published:** 2019-04-24

**Authors:** Diego O. Hartmann, Daryna Piontkivska, Carlos J. S. Moreira, Cristina Silva Pereira

**Affiliations:** Instituto de Tecnologia Química e Biológica António Xavier, Universidade Nova de Lisboa (ITQB NOVA), Oeiras, Portugal

**Keywords:** ionic liquids, cell wall, plasma membrane, sphingolipids, sphingoid base

## Abstract

Understanding stress responses and signaling pathways in fungi became a fundamental need for the discovery of new specific antifungal targets for fighting emerging life-threatening pathogens and drug resistance. Ionic liquids constitute a unique class of chemicals, which structural diversity and tunable physical and chemical properties can provide a great diversity of stimuli. In this study, we propose the use of ionic liquids as tools to unravel signaling of stress responses in the filamentous fungus *Aspergillus nidulans*. We assessed how three ionic liquids with distinct effects over the cell wall and plasma membrane affect the biosynthesis of sphingolipids and accumulation of free sphingoid bases in this fungus. The stress imposed by each ionic liquid triggered the sphingolipid biosynthetic pathway and led to distinct profiles of sphingoid bases accumulation. Dodecyltributylphosphonium chloride and 1-decyl-3-methylimidazolium chloride induced the accumulation of sphingosine and of a yet unknown sphingoid base, respectively, while cholinium decanoate did not seem to accumulate any of these intermediates. This study brings further light to the roles of sphingoid bases in *A. nidulans*. In particular, sphingosine as a possible response mediator to cell wall damage induced by dodecyltributylphosphonium chloride, and involvement of an unknown sphingoid base in the response to plasma membrane permeabilization caused by 1-decyl-3-methylimidazolium chloride. In addition, we completed the genetic assignment of the glucosylceramide pathway in *A. nidulans* through the identification of the sphingolipid Δ4-desaturase gene (*AN4405*). The knowledge established reinforces the idea of targeting sphingolipids biosynthesis in the search of improved antifungal compounds.

## Introduction

During the last few decades, fungi have been considered an increasing concern due to the rise of life-threating infections (Richardson and Warnock, 2012). Invasive fungal infections are associated with high mortality rates, killing about 1.5–2 M people every year, exceeding the deaths reported for malaria or tuberculosis (Brown et al., 2012; Denning and Bromley, 2015). The increasing number of diseases caused by fungi is mostly related to the increase of the population at risk, mainly immunocompromised individuals, such as HIV/AIDS, cancer, transplant and diabetes patients (Araujo et al., 2010; Slavin et al., 2015). Despite the growing need for efficient antifungal treatment, essentially four classes of drugs are clinically used to treat invasive fungal infections: polyenes, azoles, echinocandins and pyrimidine analogs (Vermes et al., 2000; Roemer and Krysan, 2014). The emergence of resistance to antifungal drugs is being reported more frequently, challenging the efficacy of the treatments in the near future (Kathiravan et al., 2012; Arendrup and Perlin, 2014; Perfect, 2017). Therefore, understanding of stress responses and signaling pathways in fungi became a fundamental need for discovery of new specific antifungal targets to fight antifungal drug resistance.

Ionic liquids emerge as a source of unusual and diverse tools to activate stress mechanisms in filamentous fungi. They constitute a unique class of chemicals, able to provide stimuli not often encountered in nature. Ionic liquids are generally defined as salts that are liquid below 100°C (Stark and Seddon, 2007). There are millions of possible formulations and several hundred are already well characterized (Dong et al., 2007; Stark and Seddon, 2007). Our group has for long been studying the effects of ionic liquids in filamentous fungi. Combining gene and protein expression with organic and analytical chemistry, previous studies have elucidated how particular ionic liquids affect the stress response of filamentous fungi (Petkovic et al., 2009, 2011, 2012; Petkovic and Silva Pereira, 2012; Hartmann and Silva Pereira, 2013; Martins et al., 2013; Hartmann et al., 2015, 2016; Alves et al., 2016). At sub-inhibitory concentrations, they can alter the fungal metabolic footprint (Petkovic et al., 2009), activate the biosynthesis of osmolytes (Martins et al., 2013) and uncommon secondary metabolites (Alves et al., 2016), and increase the expression of multidrug transporters (Martins et al., 2013). Recently, the effects of some 1-alkyl-3-methylimidazolium chlorides and cholinium alkanoates on the plasma membrane of *Aspergillus nidulans* have been assessed (Hartmann et al., 2015). While the first were shown to permeabilize the fungal plasma membrane, the biocompatible cholinium alkanoates did not cause this effect. Some ionic liquids have also been reported to cause damage to the cell wall of filamentous fungi (Petkovic et al., 2012; Hartmann and Silva Pereira, 2013). In yeast and filamentous fungi, damages to the cell wall can activate the cell wall integrity pathway, a response mechanism that overcomes the stress imposed over this cellular structure (Levin, 2005; Fujioka et al., 2007; Valiante et al., 2015). In *Saccharomyces cerevisiae*, cross-talk mechanisms between this pathway and other signaling pathways have been described, including the activation of the cell wall integrity pathway by sphingolipid intermediates (Levin, 2005). In *A. nidulans*, we have preliminarily observed a similar response upon exposure to ionic liquids that damage the cell wall (Hartmann and Silva Pereira, 2013, 2016; Hartmann et al., 2016).

Sphingolipids are essential components of all eukaryotic cell membranes and play an important role in a variety of biological processes, like heat stress response, signal transduction and apoptosis (Jenkins et al., 1997; Cheng et al., 2003; Hannun and Obeid, 2008). In fungi, sphingolipids such as inositol phosphorylceramides and glucosylceramides play a key role in growth and pathogenesis (Fernandes et al., 2018). Recent studies have shown that inhibition of the synthesis of these sphingolipids can attenuate the virulence of *Candida* and *Aspergillus* species (Zhong et al., 2000; Levery et al., 2002). The sphingolipid biosynthetic pathway has been characterized in greater detail in the yeast *S. cerevisiae* (Dickson and Lester, 2002) and in dimorphic fungi such as *Candida albicans* (Oura and Kajiwara, 2008) and *Cryptococcus neoformans* (Singh et al., 2012). However, knowledge on this pathway in filamentous fungi, such as *A. nidulans*, is still limited. After formation of sphingoid bases (such as dihydrosphingosine and phytosphingosine) and ceramides (Nagiec et al., 1994; Haak et al., 1997; Merrill, 2002; Singh and Del Poeta, 2016), two branches can follow up, yielding inositol phosphorylceramides or glucosylceramides. Studies have shown that the intermediates of glucosylceramide pathway can play important roles in fungal cells. In *C. albicans* disruption of sphingolipid Δ8-desaturase leads to accumulation of sphingosine and reduced hyphal growth (Oura and Kajiwara, 2008). In *C. neoformans*, mutation of the sphingolipid C9-methyltransferase gene alters the composition and rigidity of the membrane (Singh et al., 2012). *Fusarium graminearum* strains lacking the *MT2* gene, encoding a sphingolipid C9-methyltransferase, exhibit severe growth defects and produce abnormal conidia (Ramamoorthy et al., 2009). Only very recently, some of the genes of the glucosylceramide pathway in *A. nidulans* were assigned and their deletion observed to promote resistance to cell wall damaging agents, such as congo red and calcofluor white (Fernandes et al., 2016).

In the present study, we propose using ionic liquids as tools to unravel poorly characterized stress responses in *A. nidulans*. Three ionic liquids were selected – dodecyltributylphosphonium chloride ([P_4 4 4 12_]Cl), 1-decyl-3-methylimidazolium chloride ([C_10_mim]Cl) and cholinium decanoate – each displaying a distinct mechanism of toxicity (Petkovic et al., 2012; Hartmann and Silva Pereira, 2013; Hartmann et al., 2015). Toxicity evaluation, gene expression analysis by quantitative real-time PCR (*q*RT-PCR) and sphingoid base accumulation profiling by high-performance liquid chromatography (HPLC) were the main used methodologies. Collectively, the obtained data are suggestive of participation of specific sphingoid bases in the stress responses imposed by ionic liquids in either the cell wall and/or membrane of *A. nidulans*.

## Materials and Methods

### Ionic Liquids

**FIGURE 1:**
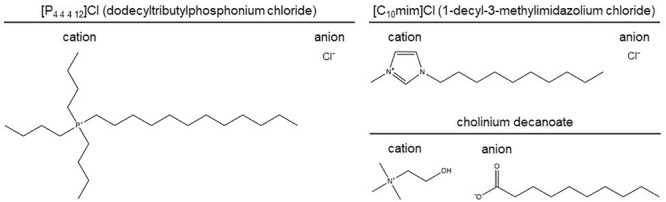
Ionic liquids used in this study.

The ionic liquids used in this study are depicted in Figure 1. Dodecyltributylphosphonium chloride, [P_4 4 4 12_]Cl, and 1-decyl-3-methylimidazolium chloride, [C_10_mim]Cl, were provided by IoLiTec Ionic Liquids Technologies, the first relying on a custom synthesis (Adamová et al., 2012). Cholinium decanoate was synthesized as previously described (Petkovic et al., 2010). Ionic liquids were characterized by ^1^H, ^13^C, and ^31^P NMR spectroscopy, mass spectrometry, CHN elemental analysis, and halide and water content analyses.

### Chemicals

The salts and supplements used to prepare the culture media were in general purchased from Sigma-Aldrich; except Dichloran-Glycerol (DG18) agar, yeast extract and bacteriological peptone that were purchased from Oxoid; sucrose from Alfa Aesar; casein hydrolysate from Fluka; agar and NaCl from Panreac AppliChem; NaOH from J.M.G. Santos and glycerol from Fisher Bioreagents. Molecular biology reagents used for PCR reactions were purchased from NZYTech. 3-(4,5-dimethylthiazol-2-yl)-2,5-diphenyl tetrazolium bromide (MTT) was purchased from Sigma-Aldrich. The reagents used for sphingolipid extraction: pyridine, butanol, ammonium hydroxide and 40% methylamine in water were purchased from Acros Organics; ethanol, methanol and chloroform (and dimethyl sulfoxide used in MTT assays) were purchased from Fisher Chemical and diethylether from Panreac AppliChem. Sphingoid bases standards (phytosphingosine, sphingosine and dihydrosphingosine) were purchased from Avanti Polar Lipids. All solvents used in chromatographic analyses were of the highest analytical grade and water was obtained from a Milli-Q system (Millipore).

### Strains and Storage Conditions

The *Aspergillus nidulans* strains used in this study are described in Supplementary Table S1. Strain A4 (used as the reference strain) and strain A1149 were obtained from the Fungal Genetics Stock Center (Kansas City, MO, United States). A1149 served as parental strain to generate Δ*AN4405*, Δ*AN4592*, Δ*AN5688*, Δ*AN7375*, Δ*AN8806* deletion mutants and a *pyrG*^+^ prototroph strain (used as control strain for experiments comparing the deletion mutants). All strains were cultivated on DG18 agar plates with the appropriate supplements. Cultures were incubated in the dark, for 6–7 days, at 30°C. Asexual spores (conidia) were harvested using a saline solution (NaCl 8.5 g⋅l^-1^) and collected after passing through three layers of miracloth. The harvested spores were resuspended in a saline solution, to be used immediately, or in a cryoprotective saline solution containing 10% (v/v) glycerol, to be stored at -20°C or -80°C.

### Experimental Conditions

Liquid minimal medium was prepared with glucose (10 g⋅l^-1^), thiamine (0.01 g⋅l^-1^), 5% (v/v) nitrate salts solution [NaNO_3_ (120.0 g⋅l^-1^), KCl (10.4 g⋅l^-1^), MgSO_4_⋅7H_2_O (10.4 g⋅l^-1^) and KH_2_PO_4_ (30.4 g⋅l^-1^)], 0.1% (v/v) trace elements solution [ZnSO_4_⋅7H_2_O (22.0 g⋅l^-1^); H_3_BO_3_ (11.0 g⋅l^-1^); MnCl_2_⋅4H_2_O (5.0 g⋅l^-1^); FeSO_4_⋅7H_2_O (5.0 g⋅l^-1^); CoCl_2_⋅6H_2_O (1.7 g⋅l^-1^); CuSO_4_⋅5H_2_O (1.6 g⋅l^-1^); Na_2_MoO_4_⋅2H_2_O (1.5 g⋅l^-1^), and Na_4_EDTA (50.0 g⋅l^-1^)] and the pH was adjusted to 6.5 with NaOH. Whenever necessary, media were supplemented with uracil (1.12 g⋅l^-1^), uridine (1.2 g⋅l^-1^), and/or pyridoxin (0.05 mg⋅l^-1^). Fungal cultures (100 ml of liquid minimal medium) were inoculated with freshly harvested spores (10^6^ conidia *per* ml) and incubated for 24 h at 30°C and 200 rpm. Ionic liquids were then added to the cultures, at the MIC, and incubated for 4 h. Longer incubation periods (8, 12, or 24 h) were used for cultures exposed to [P_4 4 4 12_]Cl. Negative controls without ionic liquids were also prepared. At the end of the treatments, mycelia were recovered by vacuum filtration and immediately frozen in liquid nitrogen. All the samples were stored at -80°C until lyophilized.

### Total RNA Extraction and *q*RT-PCR Analysis

Approximately 100 mg of frozen mycelia was grounded with poly(vinylpolypirrolidone) (0.4 mg *per* mg of mycelia) in a Tissue Lyser LT (QIAGEN). The final powder was used in the extraction and purification of total RNA using the RNeasy Plant Mini Kit (QIAGEN), according to the manufacturer’s protocol. Genomic DNA digestion was done with the RNase-Free DNase Set (QIAGEN). Quality, integrity and quantity of the total RNA were analyzed in a NanoDrop 1000 Spectrophotometer (Thermo Fisher Scientific) and by running 2 μg of RNA into 1% agarose gels. The complementary DNA (cDNA) was synthesized from 500 ng of the total RNA using an iScript cDNA Synthesis Kit (Bio-Rad) in a T100 Thermal Cycler (Bio-Rad). The reaction protocol consisted of 5 min at 25 °C, 30 min at 42°C and 5 min at 85°C. Based on the sequences of *A. nidulans* genes (*Aspergillus* Genome Database^1^), all primer pairs used in *q*RT-PCR experiments were designed using the GeneFisher2 web tool^2^, with the exception of those for *chsB, fksA, agsB*, which were previously designed in Fujioka et al. (2007). All primers were produced by Thermo Fisher Scientific (see Supplementary Table S2 for the list of the *q*RT-PCR primers used in this study). The *q*RT-PCR analysis was performed in a CFX96 Thermal Cycler (Bio-Rad), using the SsoFast EvaGreen Supermix (Bio-Rad), 250 nM of each oligonucleotide and the cDNA template equivalent to 5 ng of total RNA, at a final volume of 10 μl *per* well, in three technical replicates. The PCR conditions were: enzyme activation at 95°C for 30 s; 40 cycles of denaturation at 95°C for 10 s and annealing/extension at 59°C for 15 s; and melting curve obtained from 65°C to 95°C, consisting of 0.5°C increments for 5 s. Data analyses were performed using the CFX Manager software (Bio-Rad). The expression of each gene was taken as the relative expression compared to the time-zero (before incubation with the tested compounds). The expression of all target genes was normalized by the expression of γ-actin gene (internal control). Four biological replicates were performed. Statistical analysis of the *q*RT-PCR data was performed in the GraphPad Prism v8.0 software. Treatments with ionic liquids were compared with the control by Student’s *t*-test. Differences with a *p*-value below 0.05 were considered statistically significant.

### Free Sphingoid Base Analyses

Approximately 80 mg of dried mycelia of each condition were grounded in a Tissue Lyser LT (QIAGEN). Lipids were extracted in glass tubes with 5 ml of a solution containing 95% ethanol/water/diethylether/pyridine/4.2 M ammonium hydroxide (15:15:5:1:0.018; by volume), sonicated for 5 min and then incubated for 15 min at 60 °C. The extract was removed after centrifugation (1620 g for 5 min in a swing-bucket rotor) and the pellet extracted twice in the same manner. The lipid extracts were dried under a nitrogen flow. For methanolysis the dried extracts were resuspended in 5 ml of a solution containing methanol/ethanol/butanol/40% methylamine in water (4:3:1:4, by volume) and incubated at 52°C for 30 min to ensure the deacylation of lipids. The resulting extracts were dried under a nitrogen flow and resuspended in 3 ml of methanol/chloroform (2:1, by volume). One milliliter each of chloroform and alkaline solution (NH_4_OH in water, pH 9.0) were added to separate sphingoid bases to the organic layer. The mixture was centrifuged (1620 g for 5 min in a swing-bucket rotor), the aqueous phase was discarded and the organic phase was washed three times with 1 ml of alkaline solution, finally dried under a nitrogen flow. To perform alkaline hydrolysis the dried extracts were resuspended in 3 ml of 0.1 M KOH in methanol/chloroform (2:1, by volume) and incubated at 37°C for 1 h to break down cellular acylglycerolipids and phospholipids. After incubation, 1 ml each of chloroform and alkaline solution were added and the mixture centrifuged (1,620 g for 5 min in a swing bucket rotor). The aqueous phase was discarded and the organic phase was washed three times in the same way, then dried under nitrogen flow and finally resuspended in methanol (100 μl per each 20 mg of original dried mycelia) for further analysis of sphingoid bases by liquid chromatography. For the analysis by HPLC, 200 μl of extracts (or a 1:4 dilution for tests with glucosylceramide pathway mutants) underwent a 30 min pre-column derivatization with 25 μl *o*-phthalaldehyde solution, prepared by dissolving 5 mg *o*-phthalaldehyde and 5 μl β-mercaptoethanol in 100 μl of methanol, and adding 9.9 ml of 3% borate buffer (pH 10.5). The derivatized sphingoid bases were chromatographically separated using an Acquity UPLC System (Waters) with a fluorescence detector (acquisition with an energy gain of 1000), cooling auto sampler, and column oven. A Symmetry^®^ C18 reverse phase column (250 × 4.6 mm), packed with end capped particles (5 μm, pore size 100 Å) was used at 40°C. Data were acquired using Empower 2 software, 2006 (Waters Corporation). Sample injections of 10 μl were made using a 50 μl loop operated in partial loop mode. The mobile phase, at a flow rate of 1 ml⋅min^-1^, consisted of 90% methanol. Each sample was run for 30 min and after each run the column was washed with water. Phytosphingosine, sphingosine and dihydrosphingosine were used as standards and their chromatographic profiles can be seen in Supplementary Figures S1–S3.

### Generation of Mutant Strains

The genes *AN4405, AN4592, AN5688, AN7375*, or *AN8806* were selected for gene replacement with *A. fumigatus pyrG* in *A. nidulans* A1149 strain, performed according to a previously established method (Szewczyk et al., 2006). Generation of the prototroph *pyrG*^+^ (gene *AN6157*) was performed by the same method. Deletion cassettes were constructed using a fusion PCR protocol. For each target gene, six primers (P1-P6) were designed, based on sequences from the *Aspergillus* Genome Database and analyzed using the NetPrimer web tool^3^. All primers used for generation of gene-replacement mutants are listed in Supplementary Table S3. PCRs reactions were performed in a T100 Thermal Cycler (Bio-Rad). *A. fumigatus pyrG* was amplified from plasmid pCDS60 (FGSC, Kansas City, MO, United States) using primers CDS164 and CDS165. Flanking fragment upstream and downstream of each target gene were amplified with primer pairs P1/P3 and P4/P6, respectively, using genomic DNA from *A. nidulans* A4 as template. The final cassette was produced by fusing the flanking regions with the *A. fumigatus pyrG* using nested primers P2 and P5 for each gene target. All PCR conditions are described in Supplementary Table S4. PCR products were cleaned with NZYGelPure kit (NZYTech). To produce transformable protoplasts, 10^8^ freshly harvested conidia from *A. nidulans* A1149 were inoculated into 100 ml of liquid minimal medium with the appropriate nutritional supplements. The cultures were incubated overnight at 30°C, 90 rpm; then recovered by centrifugation and washed with 0.6 M MgSO_4_. The washed culture was resuspended in filter-sterilized enzymatic mix (300 mg lysing enzymes from *Trichoderma harzianum*, 150 μl β-glucuronidase from bovine liver, type B-1, and 150 mg Driselase from *Basidiomycetes* sp., all from Sigma-Aldrich) in osmotic medium (1.2 M MgSO_4_⋅7H_2_O and 10 mM sodium phosphate buffer pH 6.5; final pH adjusted to 5.8 with Na_2_HPO_4_) and incubated for 20 h at 30°C, 90 rpm, for digestion. Protoplasts were recovered by centrifugation and 10 ml of suspension were carefully overlaid with 5 ml of trapping buffer (0.6 M sorbitol and 100 mM Tris-HCl pH 7.0) and centrifuged at 1500 g, at 4°C, for 15 min in a swing-bucket rotor. The protoplasts phase was carefully removed and washed three times with 10 ml of ST10 buffer (1.2 M sorbitol and 10 mM Tris-HCl pH 7.5) and finally resuspended in 700 μl of STC buffer (1.2 M sorbitol, 10 mM Tris-HCl pH 7.5 and 10 mM CaCl2). For each transformation, 10 μl of cleaned fusion PCR product were added to 100 μl protoplast suspension, then vortexed shortly 6-8 times. 50 μl of freshly filtered polyethylene glycol (PEG) solution (25% (w/v) in STC buffer) were added to the mixture, vortexed 4–5 times and placed in an ice bath for 25 min. Another 1 ml of filtered PEG solution was then added, gently mixed using a micropipette and placed at room temperature for 25 min. Hundred microliter of the transformation mix were plated onto a selective medium containing glucose (5.0 g⋅l^-1^), yeast extract (5.0 g⋅l^-1^), sucrose (342.3 g⋅l^-1^), pyridoxin (0.05 mg⋅l^-1^), 0.1% (v/v) trace elements solution, 0.1% (v/v) vitamin solution [biotin (1.0 g⋅l^-1^), pyridoxin (1.0 g⋅l^-1^), thiamine (1.0 g⋅l^-1^), riboflavin (1.0 g⋅l^-1^), *p*-aminobenzoic acid (1.0 g⋅l^-1^) and nicotinic acid (1.0 g⋅l^-1^)] and 1.5% agar. The selective plates were incubated for 3–4 days at 30°C, and the isolated transformants were stroke onto complete medium plates [glucose (10.0 g⋅l^-1^), peptone (2.0 g⋅l^-1^), yeast extract (1.0 g⋅l^-1^), casein hydrolysate (1.0 g⋅l^-1^), 5% (v/v) nitrate salts solution, 0.1% (v/v) trace elements and 0.1% (v/v) vitamin solution and pyridoxine (0.05 mg⋅l^-1^), pH 6.5] and incubated at 30°C for 4 days. Three generations were obtained before DNA extraction with Quick-DNA^TM^ Fungal/Bacterial Microprep Kit (Zymo Research) and diagnostic PCR (identical protocol as fusion PCR) were done. The PCR products were digested using restriction enzymes ApaI, HpaI, BglII, or NcoI (NZYTech). Total amplification product size and differential digestion patterns allowed confirmation of correct gene replacement.

### Toxicity Tests

The minimal inhibitory concentrations (MIC) of the ionic liquids were determined as previously described (Petkovic et al., 2012). Ionic liquids were added to the minimal culture media at final concentrations ranging from 0.005 to 0.045 mM for [P_4 4 4 12_]Cl, 0.05 to 0.45 mM for [C_10_mim]Cl, and 2 to 6 mM for cholinium decanoate. For each condition, 1 ml was inoculated with 10^6^ conidia and divided into four wells (0.2 ml each) of a 96-well microtiter plate. Cultures were incubated in the dark at 30°C, for 7 days, without agitation. Fungal growth or its absence was evaluated at the end of incubation, gauging by eye the formation of mycelium (turbidity). The lowest concentration that inhibited growth was taken as the MIC. The determined values of the MIC should not be taken as absolute ones but as an indication of the upper inhibitory concentration limits. In the same conditions, the MIC was also determined for strains A4 and Δ*AN4405* in medium supplemented with exogenous sphingosine at final concentrations of 0.1, 0.5, 1.0, 2.5, 5.0, 7.5, and 10 μM. For analysis of growth in solid media, 1 μl of spore suspensions containing 10^8^, 10^7^, and 10^6^ conidia *per* ml were used to inoculate plates containing minimal media (2% agar) supplemented with [P_4 4 4 12_]Cl (0.015, 0.02, and 0.025 mM), [C10mim]Cl (0.05, 0.075, and 0.1 mM) or cholinium decanoate (0.75, 1, and 1.25 mM). Plates were incubated in the dark for 48 h at 30°C. For each condition three biological replicates were prepared.

Metabolic activity of the cultures after 4 h of exposure to each ionic liquid was verified by the reduction of 3-(4,5-dimethylthiazol-2-yl)-2,5-diphenyl tetrazolium bromide (MTT). *Aspergillus nidulans* (10^6^ conidia *per* ml) was grown in minimal medium for 24 h, 30°C, without agitation, in 96-well plates, at a final volume of 200 μl *per* well (six wells *per* condition). The medium was carefully removed and 200 μl of fresh medium supplemented with either ionic liquid at the MIC concentration (only minimal medium for the control) was added to each well and incubated at 30°C for 4 h. The medium was then removed and the mycelia washed three times with saline. Hundred microliter of fresh minimal medium and 20 μl of a freshly prepared stock of MTT (5 mg⋅ml^-1^ in saline) were then added to each well and plates were incubated at 30°C, in the dark, for 4 h. The supernatant was then aspirated and 100 μl of dimethyl sulfoxide was added to each well to dissolve the resulting formazan crystals, overnight, in the dark. The plates were read in a Tecan Infinite M200 spectrophotometer, scoring the absorbance at 570 nm. The values of metabolic activity for each ionic liquid were obtained relatively to the control. Four biological replicates were performed.

### Microscopic Analyses

*Aspergillus nidulans* (10^3^ conidia *per* ml) was cultivated in 1 ml minimal medium in 12-well plates and grown for 24 h, at 30°C, without agitation. Prior to incubation plates were centrifuged at 600 g for 5 min to sediment conidia to the bottom of the wells. After growth, medium was carefully aspirated from each well and 1 ml of fresh medium supplemented with 100 mM of each ionic liquid was added. The plates were incubated for 4 h. Medium without supplementation was added for the control. After incubation, the medium from each well was carefully removed and the adhered mycelia were washed four times with a saline solution. Mycelia were stained with 1 ml calcofluor white, at the final concentration of 25 μM, and incubated at 30°C, for 30 min, in the dark. Residual dye was removed by carefully washing the mycelia with saline solution three times. The stained mycelia were visualized in a Nikon Eclipse Ts2-FL inverted fluorescence microscope with a 20× magnification and a C-LED 385 nm filter. Images were captured with a Nikon DS-Fi3 camera. This assay provided a qualitative analysis of the alterations provoked by exposure to the ionic liquids in the hyphal cell wall.

## Results

### [P_4 4 4 12_]Cl Induces Sphingolipid Biosynthesis and Accumulation of Sphingosine

**FIGURE 2:**
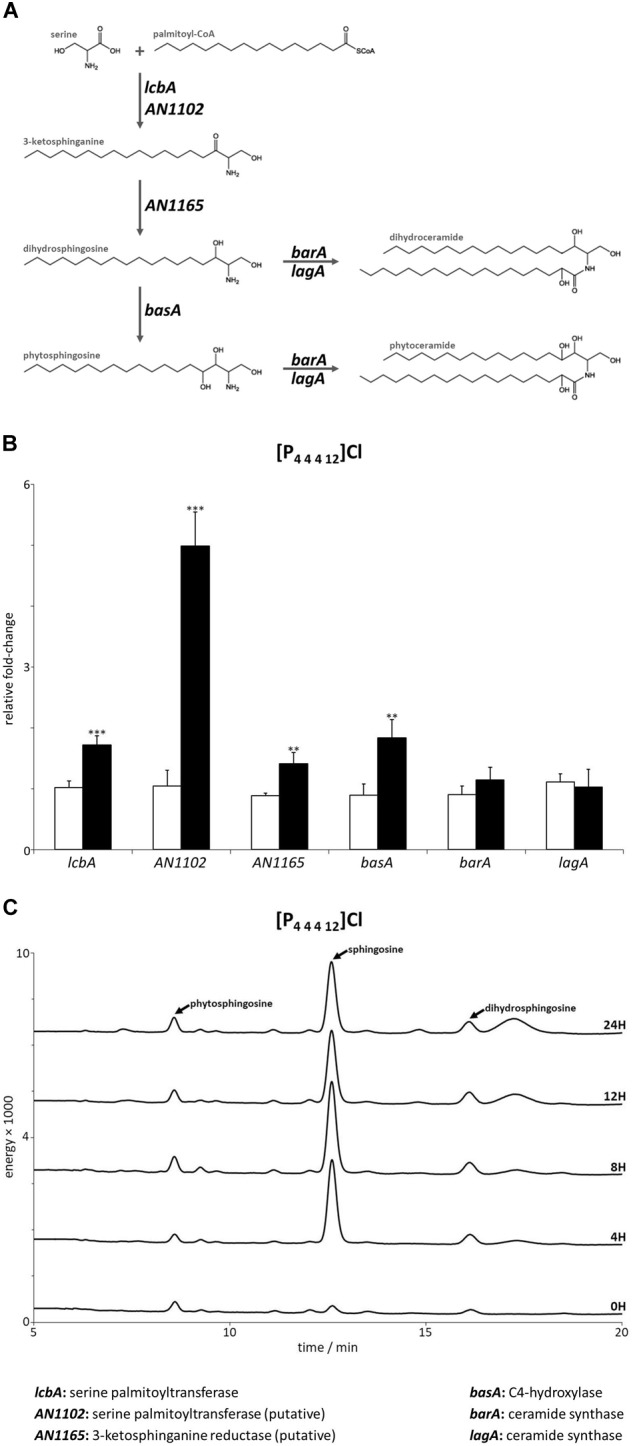
**(A)** Schematic representation of the first steps in the sphingolipid biosynthetic pathway. **(B)** Expression levels of the genes mediating the first steps of sphingolipid biosynthesis after 4 h of exposure to dodecyltributylphosphonium chloride ([P_44412_]Cl, black bars) compared to the control (white bars). Axis y represents the fold-change relative to time-zero (^∗∗^*p* < 0.01, ^∗∗∗^*p* < 0.001). **(C)** Sphingoid bases accumulation profile after 4, 8, 12, and 24 h of exposure to [P_44412_]Cl. The peaks assigned to phytosphingosine, sphingosine and dihydrosphingosine were confirmed by their standards.

In previous reports, we have demonstrated that alkyltributylphosphonium chlorides, ionic liquids recalcitrant to degradation, damage the cell wall of *A. nidulans* and cause membrane permeabilization when a long alkyl substituent is present (Petkovic et al., 2012; Hartmann and Silva Pereira, 2013). In this study, we tested whether the stress imposed by [P_4 4 4 12_]Cl – an ionic liquid from this family that has an alkyl substituent composed of 12 carbon atoms (Figure 1) – could alter the biosynthesis of sphingolipids. After 4 h of exposure to [P_4 4 4 12_]Cl at the MIC (Table 1), *A. nidulans* cultures remained metabolically active (Supplementary Table S5) and the expression of genes involved in sphingolipid biosynthesis was further analyzed by *q*RT-PCR. A schematic representation of the sphingolipid biosynthetic pathway is depicted in Figure 2A (Levin, 2005; Li et al., 2006, 2007). Gene expression data suggested that the sphingolipid biosynthetic pathway was activated upon exposure to [P_4 4 4 12_]Cl. In detail, *lcbA*, the gene coding for a serine palmitoyltransferase that catalyzes the very first step in the biosynthesis of sphingoid bases (Cheng et al., 2001), underwent a significant 1.7-fold increase in its expression after exposure to the ionic liquid (Figure 2B and Supplementary Table S7). It is likely that, as in *S. cerevisiae*, this enzyme is comprised of two components (LCB1 and LCB2) (Nagiec et al., 1994). *Aspergillus nidulans lcbA* is homolog to the yeast *LCB1*. Although not described, *AN1102* has very high similarity with *LCB2*, and putatively encodes another component of the serine palmitoyltransferase complex in *A. nidulans* (Supplementary Table S6). After exposure to [P_4 4 4 12_]Cl, *AN1102* expression increased fivefold (Figure 2B and Supplementary Table S7). The up-regulation of both genes is consistent with the activation of sphingolipid biosynthesis under the ionic liquid stress.

**Table 1:** Minimal inhibitory concentrations (in mM) of the tested ionic liquids against *Aspergillus nidulans* strains after growth in liquid minimal medium.

Strain	[P_44412_]Cl	[C_10_mim]Cl	Cholinium decanoate
A4	0.015	0.24	3.0
A1149 *pyrG*^+^	0.015	0.24	3.0
Δ*AN4405*	0.012	0.10	2.7
Δ*AN4592*	0.013	0.11	2.4
Δ*AN5688*	0.012	0.19	3.4
Δ*AN7375*	0.010	0.21	3.3
Δ*AN8806*	0.011	0.17	2.5

The following steps of the sphingolipid biosynthetic pathway are involved in the formation of the sphingoid bases dihydrosphingosine and phytosphingosine (Figure 2A). These steps are catalyzed by enzymes encoded by *AN1165* (putatively, Supplementary Table S6) (Beeler et al., 1998) and *basA* (Li et al., 2007), respectively. Both genes were significantly up-regulated 1.4 and 1.8-fold upon [P_4 4 4 12_]Cl stress compared to the control (Figure 2B and Supplementary Table S7). Interestingly, the synthesis of ceramides, the next step in the pathway, did not seem to be triggered, as inferred by the unaltered expression of the genes coding for ceramide synthases *barA* and *lagA* (Figure 2B and Supplementary Table S7) (Li et al., 2006). Collectively, these data expose that the stress imposed by [P_4 4 4 12_]Cl activated the genes involved in the synthesis of sphingoid bases but not ceramides, likely leading to accumulation of sphingoid bases.

To further confirm the accumulation of free sphingoid bases, they were extracted from mycelia of *A. nidulans* after exposure to [P_4 4 4 12_]Cl for 4, 8, 12, or 24 h and analyzed by liquid chromatography after derivatization with *o*-phthalaldehyde. The obtained chromatograms clearly reveal that the ionic liquid stress stimulated the accumulation of the sphingoid base sphingosine as early as 4 h, maintained up to 24 h of exposure to the ionic liquid (Figure 2C).

### [P_4 4 4 12_]Cl Stress Response Involves the Glucosylceramide Biosynthetic Pathway in *Aspergillus nidulans*

**FIGURE 3:**
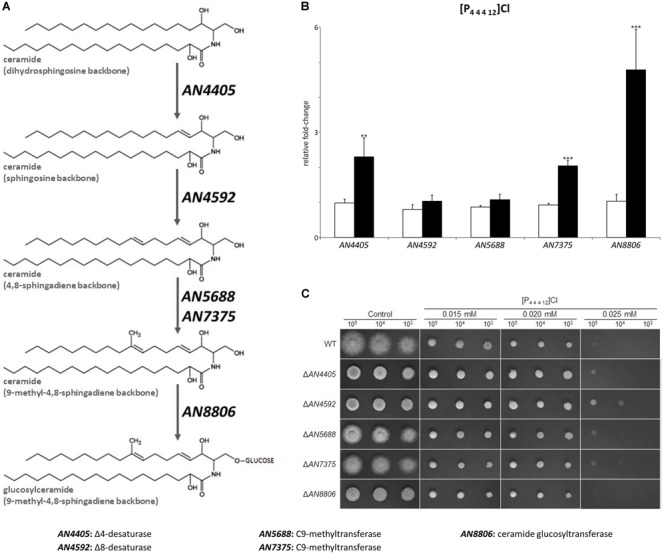
**(A)** Schematic representation of the glucosylceramide pathway. **(B)** Expression analysis of glucosylceramide pathway genes after 4 h of exposure to dodecyltributylphosphonium chloride ([P_44412_]Cl, black bars), compared to the control (white bars). Axis y represents the fold-change relative to time-zero (^∗∗^*p* < 0.01, ^∗∗∗^*p* < 0.001). **(C)** Growth assessment in solid media supplemented with 0.015, 0.020, or 0.025 mM of [P_44412_]Cl. 10^5^, 10^4^, and 10^3^ conidia were inoculated in each plate.

As known for other fungal species, sphingosine is synthesized through the glucosylceramide branch of sphingolipid biosynthesis (schematic representation in Figure 3A) (Del Poeta et al., 2014). In *A. nidulans*, part of the branch is known, namely Δ8-desaturase (*AN4592*), C9-methyltransferases (*AN5688* and *AN7375*) and ceramide glucosyltransferase (*AN8806*) genes (Fernandes et al., 2016). However, the gene involved in the synthesis of sphingosine (i.e., coding for a sphingolipid Δ4-desaturase) remains unknown. To attempt its identification, the sequence of the previously verified dihydroceramide Δ4-desaturase from *Schizosaccharomyces pombe* (encoded by *dsd1*) (Garton et al., 2003) was used as query in protein-protein BLAST analysis (Supplementary Table S6). The best obtained hit was *A. nidulans AN4405* gene (*e* = 6.0e^-4^, 93.0% query coverage, 54.0% identity), consistent with the predicted functions described in the *Aspergillus* Genome Database. The *AN4405* gene model is located at chromosome III and comprises 1381 nucleotides with two predicted introns from 211 to 273 and 553 to 613; the open reading frame contains 1257 bp encoding 418 amino acids. A gene-replacement mutant (Δ*AN4405*) was generated and its sphingoid base accumulation profile after 24 h of growth was analyzed by liquid chromatography, further confirming its assignment as the gene coding for a sphingolipid Δ4-desaturase in *A. nidulans* (Supplementary Figure S4).

The expression of genes involved in the glucosylceramide pathway after exposure to 4 h of [P_4 4 4 12_]Cl were also analyzed. Concordant to the observed accumulation of sphingosine, *AN4405* underwent a significant increase of 2.3-fold compared to the control, while the gene responsible for the following step in the pathway, *AN4592*, did not alter its expression (Figure 3B and Supplementary Table S7). To additionally assess sphingosine involvement in the stress response imposed by [P_4 4 4 12_]Cl, the MIC values of wild-type and Δ*AN4405* strains when exogenous sphingosine was added to the growth media were determined. Data show that increasing concentrations of exogenous sphingosine enabled Δ*AN4405* to tolerate higher amounts of [P_4 4 4 12_]Cl, reaching values above the original wild-type MIC when 5 μM or more were present in the medium (Table 2). The wild-type strain was also able to increase its tolerance to the ionic liquid in medium with 2.5 μM or more of exogenous sphingosine.

**Table 2:** Minimal inhibitory concentrations (in mM) for [P_44412_]Cl against *Aspergillus nidulans* strains A4 and Δ*AN4405* after growth in liquid minimal medium supplemented with different concentrations of exogenous sphingosine (in μM).

Strain	Sphingosine concentration (μM)							
	0	0.1	0.5	1.0	2.5	5.0	7.5	10.0
A4	0.015	0.015	0.015	0.015	0.017	0.019	0.019	0.019
Δ*AN4405*	0.012	0.012	0.012	0.012	0.013	0.017	0.018	0.018

To further evaluate the involvement of the glucosylceramide pathway in the stress response imposed by [P_4 4 4 12_]Cl, we generated gene-replacement mutants for the remaining genes of the glucosylceramide pathway and evaluated their susceptibility to the ionic liquid treatment. The determined MICs reveal higher susceptibility of all the mutants to [P_4 4 4 12_]Cl compared to the wild-type strain (Table 1), consistent with the involvement of the glucosylceramide pathway in the stress response. When looking at the growth on solid media, only the strain Δ*AN4592* could grow well when 10^5^ and 10^4^ spores were inoculated in plates at the highest tested concentration of [P_4 4 4 12_]Cl (0.025 mM) (Figure 3C). Since this mutant is unable to utilize ceramides with a sphingosine backbone, it is likely that Δ*AN4592* accumulates free sphingosine, reinforcing the potential role of this sphingoid base in signaling the stress induced by the ionic liquid.

### Ionic Liquids With Distinct Effects on the Cell Wall and Plasma Membrane Also Activate the Sphingolipid Biosynthetic Pathway

**FIGURE 4:**
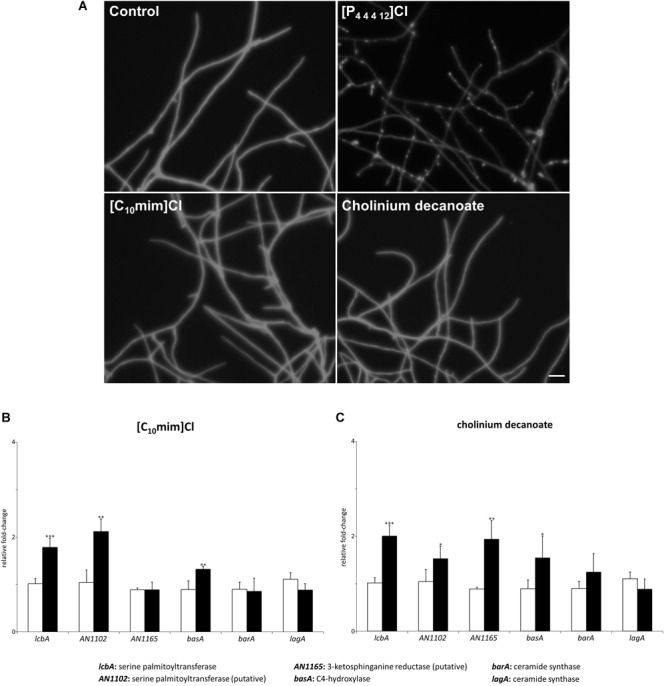
**(A)** Microscopic images of hyphae exposed to 100 mM of dodecyltributylphosphonium chloride ([P_44412_]Cl), 1-decyl-3-methylimidazolium chloride ([C_10_mim]Cl) or cholinium decanoate for 4 h and stained with calcofluor white (scale bar: 25 μM). **(B,C)** Expression levels of the genes mediating the first steps of sphingolipid biosynthesis after 4 h of exposure to [C_10_mim]Cl (**B**, black bars) or cholinium decanoate (**C**, black bars) compared to the control (white bars). Axis y represents the fold-change relative to time-zero (^∗^*p* < 0.05, ^∗∗^*p* < 0.01, ^∗∗∗^*p* < 0.001).

As observed above, [P_4 4 4 12_]Cl, which is able to cause damage to both the cell wall and plasma membrane of *A. nidulans* (Petkovic et al., 2012; Hartmann and Silva Pereira, 2013), activated sphingolipid biosynthesis and induced the accumulation of sphingosine. We decided to test if ionic liquids with distinct effects would also affect the sphingolipid biosynthetic pathway in *A. nidulans*. Two ionic liquids, representative of families that we previously reported to have opposing effects over the plasma membrane (Hartmann et al., 2015), were selected: [C_10_mim]Cl (long alkyl substituent in the cation and recalcitrant to degradation) that permeabilizes the plasma membrane; and cholinium decanoate (long alkyl anion and biodegradable) that does not induce the same effect on the lipid bilayer. Similarly to [P_4 4 4 12_]Cl, after 4 h of exposure to either ionic liquid at the MIC, *A. nidulans* cultures were still metabolically active (Supplementary Table S5). We observed by microscopy that, unlike [P_4 4 4 12_]Cl, neither [C_10_mim]Cl or cholinium decanoate caused cell wall damage in *A. nidulans* hyphae after 4 h of exposure to 100 mM of ionic liquid (Figure 4A). This was further confirmed by *q*RT-PCR analysis of genes involved in the biosynthesis of chitin (*chsA, chsB, chsC, chsD, chsF, chsG, csmA*, and *csmB*), α-glucans (*agsA, agsB*) and β-glucans (*fksA*) after 4 h of exposure to each ionic liquid (Table 3). While the cell wall damaging [P_4 4 4 12_]Cl led to a significant increase in most tested cell wall biosynthetic genes, neither [C_10_mim]Cl or cholinium decanoate altered their expression (Table 3 and Supplementary Table S7). The exceptions were *chsD* and *fksA*, that presented an increase of 1.6- and 1.3-fold (*chsD*) and 1.6- and 1.7-fold (*fksA*) for [C_10_mim]Cl and cholinium decanoate, respectively. These values are nonetheless much lower than those obtained after exposure to [P_4 4 4 12_]Cl: 3.2-fold increase for *chsD* and 6.6-fold for *fksA*. Altogether, the data revealed, as expected, that each ionic liquid induce a distinct stress over *A. nidulans*, yet the activation of the sphingolipid biosynthetic pathway was observed for all (Figure 2B, 4B,C). Exposure to [C_10_mim]Cl significantly increased the expression of *lcbA, AN1102*, and *basA* 1.8-, 2.1-, and 1.3-fold, respectively (Figure 4B and Supplementary Table S7). Cholinium decanoate increased *lcbA, AN1102, AN1165*, and *basA* 2.0-, 1.5-, 1.9-, and 1.6-fold, respectively (Figure 4C and Supplementary Table S7). As observed for [P_4 4 4 12_]Cl, the expression of genes involved in ceramide synthesis (*barA* and *lagA*) remained unaltered (Figure 4B,C and Supplementary Table S7), suggesting that the stress imposed by [C_10_mim]Cl or cholinium decanoate also led to the accumulation of sphingoid bases.

**Table 3:** Gene expression analysis (*q*RT-PCR) of genes involved in the synthesis of chitin, α-glucans and β-glucans after exposure to dodecyltributylphosphonium chloride ([P_4 4 4 12_]Cl), 1-decyl-3-methylimidazolium chloride ([C_10_mim]Cl) or cholinium decanoate for 4 h.

Gene	Control	[P_44412_]Cl	[C_10_mim]Cl	Cholinium decanoate
*chsA*	0.97	**1.37**	0.76	1.03
*chsB*	1.14	**3.01**	1.71	1.73
*chsC*	0.90	**0.46**	**0.48**	1.18
*chsD*	1.08	**3.22**	**1.60**	**1.33**
*chsF*	1.04	**0.56**	0.86	0.95
*chsG*	1.02	1.11	1.04	0.89
*csmA*	0.92	**3.11**	1.16	1.72
*csmB*	0.98	**1.24**	1.04	1.21
*agsA*	0.86	**2.04**	0.91	1.13
*agsB*	0.91	**1.33**	1.03	1.29
*fksA*	1.06	**6.58**	**1.56**	**1.65**

*Values represent the fold-change relative to time-zero followed by their standard deviation. Four biological replicates were performed. Values in bold are statistically significant compared to the control (p < 0.05).*

### The Stress Response Imposed by [C_10_mim]Cl Involves the Accumulation of an Unknown Sphingoid Base

**FIGURE 5:**
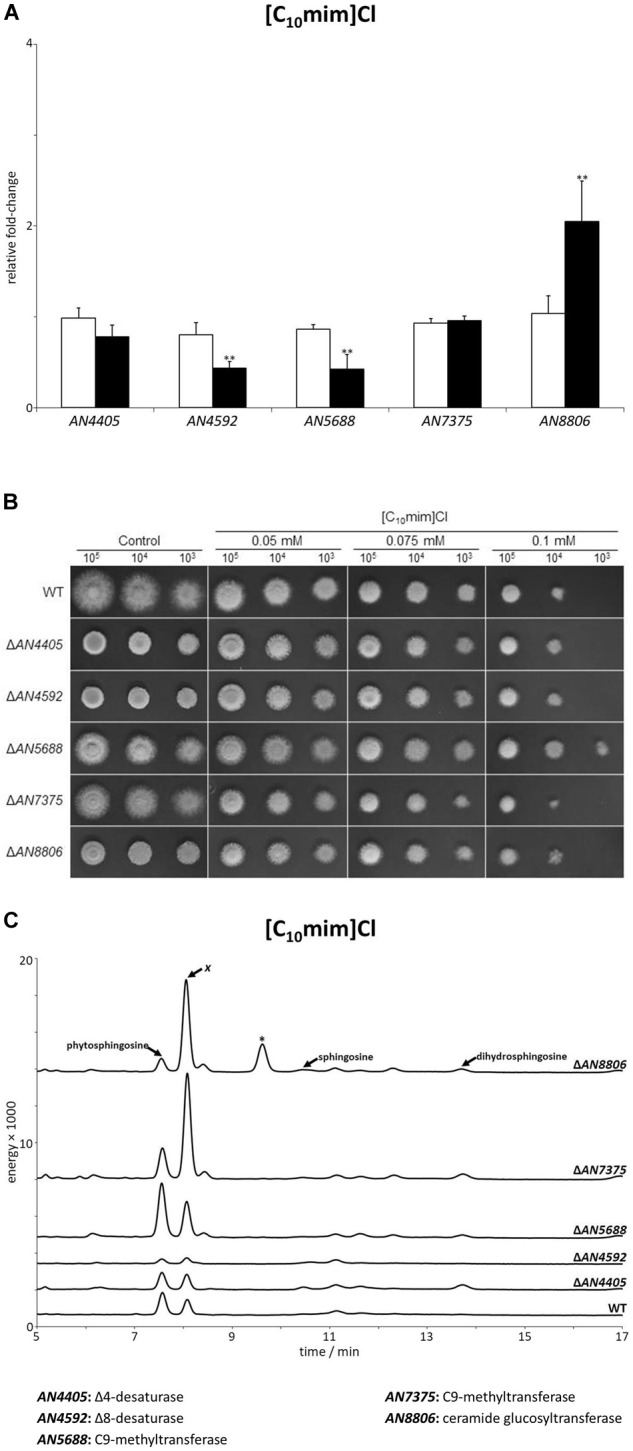
**(A)** Expression analysis of glucosylceramide pathway genes after 4 h of exposure to 1-decyl-3-methylimidazolium chloride ([C_10_mim]Cl, black bars), compared to the control (white bars). Axis y represents the fold-change relative to time-zero (^∗∗^*p* < 0.01). **(B)** Growth assessment in solid media supplemented with 0.05, 0.075, or 0.1 mM of [C_10_mim]Cl. 10^5^, 10^4^, and 10^3^ conidia were inoculated in each plate. **(C)** Sphingoid bases accumulation profile of glucosylceramide pathway mutants and parental strain (wt) after 4 h of exposure to [C_10_mim]Cl. The peaks assigned to phytosphingosine, sphingosine and dihydrosphingosine were confirmed by their standards. The peak marked with an asterisk likely corresponds to 9-methyl-4,8-sphingadiene (more details in Supplementary Figure S5).

We additionally examined the alterations in expression of the glucosylceramide pathway genes after 4 h of exposure to the plasma membrane permeabilizing ionic liquid [C_10_mim]Cl. Only *AN8806* had an increase (twofold) in its expression, while no increase was observed for *AN4405, AN4592, AN5688*, or *AN7375* (Figure 5A and Supplementary Table S7). In fact, *AN4592* and *AN5688* significantly decreased their expression after exposure to the ionic liquid. Mutant strains of the glucosylceramide pathway displayed a higher susceptibility to the ionic liquid, as seen by lower MIC values compared to the wild-type strain (Table 1). However, when grown on solid media supplemented with [C_10_mim]Cl, all mutant strains showed similar susceptibility to the wild type, even at the highest concentration tested (Figure 5B), except Δ*AN5688 –* the only that grow when 10^3^ conidia where inoculated in plates containing 0.1 mM of the ionic liquid. To further understand these differences, we assessed the sphingoid bases accumulation profile of the wild-type and mutant strains confronted with [C_10_mim]Cl for 4 h. The obtained chromatograms revealed that, unlike when *A. nidulans* was exposed to [P_4 4 4 12_]Cl (Figure 2C), no accumulation of sphingosine was noticed (Figure 5C). The lack of sphingosine accumulation is consistent with the observed unaltered expression of *AN4405* after exposure to [C_10_mim]Cl (Figure 5A). Remarkably, wild-type and all mutant strains accumulated an unknown sphingoid base *x* (Figure 5C), which shows a distinct chromatographic eluting profile from phytosphingosine, sphingosine or dihydrosphingosine. The presence of sphingoid base *x* in all strains strongly suggests that its formation is independent of the glucosylceramide pathway. Nevertheless, the amounts accumulated of sphingoid base *x* were apparently correlated with the susceptibility of the mutant strains to the ionic liquid: Δ*AN4405* and Δ*AN4592* accumulated lower amounts and were more susceptible; and Δ*AN5688*, Δ*AN7375* and Δ*AN8806* accumulated higher amounts and were less susceptible. This suggests a potential role of sphingoid base *x* in signaling the membrane damage stress imposed by [C_10_mim]Cl.

### Stress Response Provoked by Cholinium Decanoate Does Not Involve the Accumulation of Sphingosine or Sphingoid Base *x*

**FIGURE 6:**
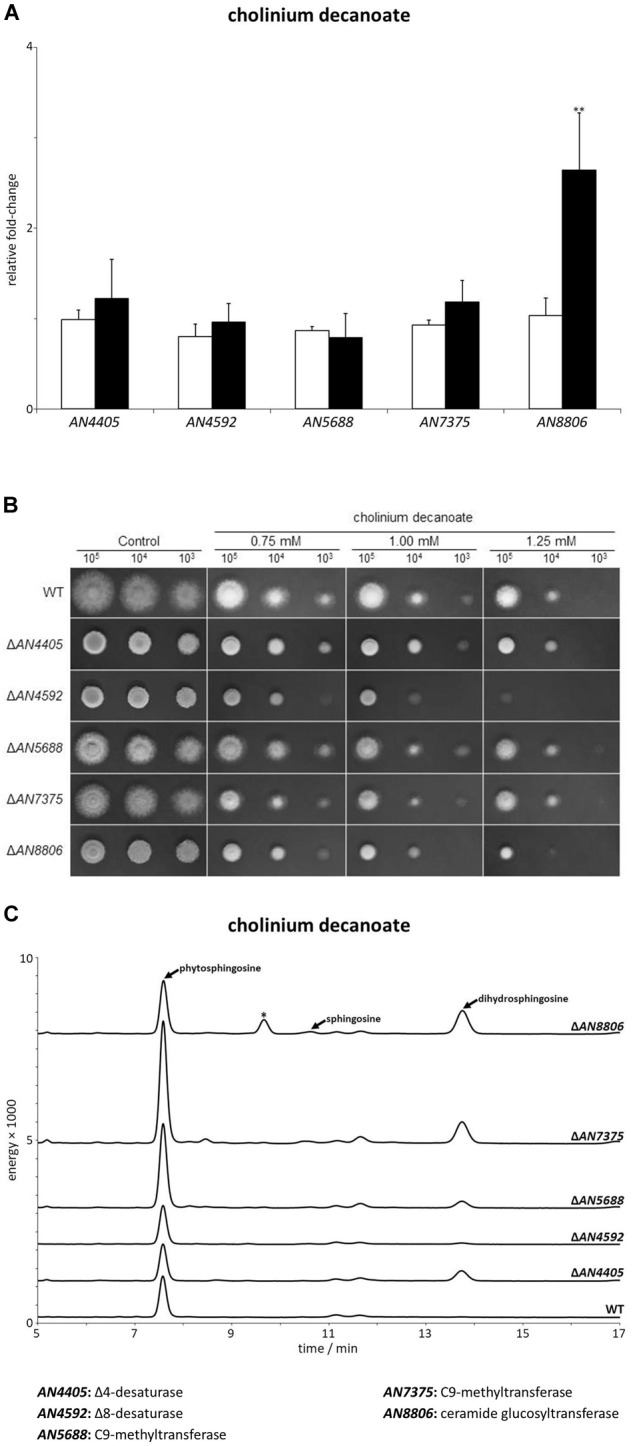
**(A)** Expression analysis of glucosylceramide pathway genes after 4 h of exposure to cholinium decanoate (black bars), compared to the control (white bars). Axis y represents the fold-change relative to time-zero (^∗∗^*p* < 0.01). **(B)** Growth assessment in solid media supplemented with 0.75, 1.0, or 1.25 mM of cholinium decanoate. 10^5^, 10^4^, and 10^3^ conidia were inoculated in each plate. **(C)** Sphingoid bases accumulation profile of glucosylceramide pathway mutants and parental strain (wt) after 4 h of exposure to cholinium decanoate. The peaks assigned to phytosphingosine, sphingosine and dihydrosphingosine were confirmed by their standards. The peak marked with an asterisk likely corresponds to 9-methyl-4,8-sphingadiene (more details in Supplementary Figure S5).

The last ionic liquid tested, cholinium decanoate, stands out from [P_4 4 4 12_]Cl and [C_10_mim]Cl due to its inability to cause plasma membrane permeabilization or cell wall damage (Figure 4A and Table 3), yet apparently also activating the sphingolipid biosynthetic pathway (Figure 4C). After 4 h of exposure to cholinium decanoate, only the *AN8806* gene among those of the glucosylceramide pathway increased its expression, namely 2.6-fold (Figure 6A). Strains Δ*AN4405*, Δ*AN4592*, and Δ*AN8806* were more susceptible to cholinium decanoate than the wild-type strain, whereas Δ*AN5688* and Δ*AN7375* presented a slightly higher tolerance (Table 3). A similar pattern was observed in solid media supplemented with the ionic liquid (Figure 6B). Strains Δ*AN4592* and Δ*AN8806* were unable to grow when 10^3^ conidia were inoculated in plates containing 1 mM of cholinium decanoate (Figure 6B). On the other hand, we could observe some initial development of colonies for strains Δ*AN5688* and Δ*AN7375* when 10^3^ conidia were inoculated on plates with the highest concentration of the ionic liquid (Figure 6B). When looking at the sphingoid base accumulation profile after 4 h of exposure to cholinium decanoate, we observe that strains with higher tolerance (Δ*AN5688* and Δ*AN7375*) apparently presented high amounts of phytosphingosine.

## Discussion

In this work, we analyzed the response of *A. nidulans* to the stress induced by three ionic liquids, namely [P_4 4 4 12_]Cl, [C_10_mim]Cl and cholinium decanoate. Despite the distinct stresses imposed by each ionic liquid, we observed by gene expression analysis that all tested ionic liquids were able to activate the sphingolipid biosynthetic pathway, as seen by the increased expression of genes *lcbA, AN1102, AN1165*, and *basA* (Figure 2B, 4B,C). The accumulation of free sphingoid bases was also suggested due to the unaltered expression of the genes involved in synthesis of ceramides (*barA* and *lagA*). The gene expression data were validated by the chromatographic analysis of the ensuing sphingolipid extracts. Accumulation of free sphingoid bases upon the stress imposed by the ionic liquids could be a strong indicative of their role as signaling molecules. The lipid extraction protocol used in this study includes a mild alkaline hydrolysis that does not break down complex sphingolipids (Li et al., 2006). Consequently, only the free sphingoid bases are extracted but not those that are part of ceramides and of complex sphingolipids that make up cellular membranes. Both [P_4 4 4 12_]Cl and [C_10_mim]Cl led to a clear accumulation of specific free sphingoid bases: sphingosine (Figure 2C) and the unknown sphingoid base *x* (Figure 5C), respectively, none of which accumulated when cholinium decanoate was used instead (Figure 6C).

Upon exposure to [P_4 4 4 12_]Cl, *A. nidulans* accumulated sphingosine, suggesting involvement of this sphingoid base in the stress response to cell wall damage, the primary mechanism of toxicity of this family of ionic liquids (Petkovic et al., 2012; Hartmann and Silva Pereira, 2013). It has already been shown in *S. cerevisiae* that the cell wall integrity pathway can be regulated by the sphingolipid biosynthetic pathway through indirect activation of protein kinase Pkc1 by phytosphingosine (Levin, 2005). This sphingoid base has been demonstrated to be a lipid activator of protein kinase Pkh1 that in turn regulates downstream kinases including Ypk1/2 and Pkc1, which control cell wall integrity and growth, among other processes (Liu et al., 2005). Our data suggest that response to cell wall stress in *A. nidulans* involves the participation of sphingosine, instead of phytosphingosine. Supporting this, it has been observed that in mammals, PDK1, protein homologous to yeast Pkh1 can be activated by sphingosine (King et al., 2000). Indeed, we observed that addition of exogenous sphingosine to the growth medium increased the tolerance of the wild-type strain to [P_4 4 4 12_]Cl, reaching values above the original MIC (Table 2).

In fungi, sphingosine is produced in the first step of the glucosylceramide pathway (Del Poeta et al., 2014), catalyzed by a sphingolipid Δ4-desaturase. Although the following steps of the pathway have been recently identified (Fernandes et al., 2016), up to now the gene responsible for the sphingolipid Δ4-desaturase function remained uncharacterized. We generated the deletion mutant for gene *AN4405*, homolog to *S. pombe dsd1* (Garton et al., 2003). This strain was unable to produce sphingosine and accumulated dihydrosphingosine (the precursor sphingoid base), as observed by liquid chromatography (Supplementary Figure S4). This identification is further confirmed by the increased expression of gene *AN4405* by the wild-type strain exposed to [P_4 4 4 12_]Cl, which accumulates sphingosine (Figure 2C, 3B). Supplementation of the growth medium with exogenous sphingosine restored the ability of the mutant strain Δ*AN4405* to tolerate higher concentrations of the ionic liquid, at levels similar to the wild-type (Table 2). Accumulation of sphingosine appears to be indeed related to the cell wall stress response in *A. nidulans*.

Imidazolium-based ionic liquids have been frequently reported to be toxic toward many organisms, from bacteria to higher multicellular ones (Petkovic et al., 2011). This is likely due to their ability to interact with and permeabilize the plasma membrane, a cellular structure common to all these organisms. Molecular dynamic simulations proposed that the imidazolium cations interact and are inserted into the lipid bilayer, with the imidazolium ring placed along with the lipid heads and the alkyl chain deep into the hydrophobic portion (Yoo et al., 2014). This incorporation not only induces structural damage to the bilayer, but also impact on the activity of membrane proteins and ions flux (Ryu et al., 2015). These have also been investigated in model lipid membranes (Galluzzi et al., 2013) and diverse living organisms, such as fungi (Hartmann et al., 2015) and worms (Charan et al., 2015). When analyzing the effects of exposure to [C_10_mim]Cl no accumulation of sphingosine was observed. None of the strains, either wild-type or impaired in one of the steps of the glucosylceramide pathway, have shown accumulation of this sphingoid base. However, they all presented increase production of an unknown sphingoid base *x* (Figure 5C). The response to the stress imposed over the plasma membrane likely involves sphingoid base *x*, since higher its accumulation, lesser the strain susceptibility to [C_10_mim]Cl. We have attempted the identification of this unknown sphingoid base by mass spectrometry but a precise assessment was not possible since this compound, which is present in low levels, was masked by fatty acids (data not shown). The liquid chromatographic method used is blind to fatty acids since the sphingoid bases are only visible after the derivatization of the primary amines (Merrill et al., 2000).

The sphingoid base *x* elutes after phytosphingosine, hence we believe its formation involves modifications in the dihydrosphingosine or sphingosine backbone, e.g., addition of a double bond distinct from the Δ8-desaturation; this hypothesis will be further analyzed in the near future. The diversity of sphingoid bases already identified in plants includes compounds, e.g., formed through the addition of double bonds in the phytosphingosine backbone (Markham et al., 2006). Moreover, unusual modifications such as the presence of a Δ10-desaturation in a 4,8-sphingadiene backbone has been observed in the diatom *Thalassiosira pseudonana* (Michaelson et al., 2013) and, more recently, in *F. graminearum* (Tian et al., 2015). These recent reports reinforce the idea that uncommon structural modifications may exist in *A. nidulans*.

The last tested ionic liquid, cholinium decanoate, apparently did not provoke accumulation of any sphingoid base (Figure 6C). Although great amounts of phytosphingosine were present in the strains with higher tolerance to the ionic liquid (Δ*AN5688* and Δ*AN7375*), higher amounts of this sphingoid base were also noticed in these mutant strains without any stress imposition (Supplementary Figure S5). Strain Δ*AN4592* revealed to be the most susceptible to the ionic liquid stress, although no correlation could be made with the sphingoid base accumulation profile. It is possible that downstream effectors, not seen by the methodologies employed, could be involved in this increased susceptibility. Deeper understanding of the ionic liquid toxicity could help solving this question. Previous studies have shown that *A. nidulans* decreases cholinium decanoate concentrations in the growth medium along time (Hartmann et al., 2015) and that filamentous fungi can uptake the cholinium cation (Markham et al., 1993). A proteomic study by our group shed light on the mechanism of toxicity of cholinium-based ionic liquids (Martins et al., 2013). It was shown that major uptake of the cholinium cation led to the production of the toxic compound cyanide, which might be a cause of the observed growth inhibition provoked by this group of ionic liquids (Martins et al., 2013). Upon exposure to cholinium decanoate, neither sphingosine nor sphingoid base *x* were accumulated, further supporting the involvement and specificity of these sphingoid bases in signaling the stress imposed by cell wall damage or plasma membrane permeabilization, respectively.

## Conclusion

Sphingoid bases – intermediates of the sphingoid biosynthetic pathway – have been demonstrated to play significant roles as signaling molecules in fungi, yet many aspects remain poorly understood. Our study sheds light to the roles of specific sphingoid bases in stress responses, namely sphingosine functioning in response to cell wall damage and of a yet unknown sphingoid base (possibly formed from a sphingosine or dihydrosphingosine backbone) in response to membrane damage. In addition, we also fill one important gap in the genetic assignment of the glucosylceramide pathway in *A. nidulans* through the identification of the sphingolipid Δ4-desaturase gene (*AN4405*). Our findings were made possible through the use of ionic liquids – compounds yet largely unexplored in biological sciences. Nonetheless, the structural diversity and tunable physical and chemical properties of these classes of compounds may provide a diversity of useful stimuli, such as cell wall damage, plasma membrane permeabilization and particular alterations in metabolism, to deepen our understanding of stress signaling responses in fungi. It comes without saying that the knowledge established reinforces the idea of targeting sphingolipids biosynthesis for the search of improved antifungal compounds.

## Author Contributions

DH contributed to planning, execution and analysis of all experiments. DP contributed to generation of mutants and respective experiments. CM contributed to experiments with dodecyltributylphosphonium chloride and wild-type strain. DH and CSP wrote the manuscript. All authors reviewed the results and approved the final version of the manuscript.

## Conflict of Interest Statement

The authors declare that the research was conducted in the absence of any commercial or financial relationships that could be construed as a potential conflict of interest.

## Footnotes

1 http://www.aspergillusgenome.org

2 http://bibiserv.techfak.uni-bielefeld.de/genefisher2

3 http://www.premierbiosoft.com/NetPrimer/AnalyzePrimer.jsp
