# Supplementary material for: Ionic Liquids Chemical Stress Triggers Sphingoid Base Accumulation in Aspergillus nidulans
Source: Front Microbiol. 2019 Apr 24;10:864. doi: 10.3389/fmicb.2019.00864 (PMC6491925; doi:10.3389/fmicb.2019.00864)
Supplement: Supplementary file 1 [file Data_Sheet_1.PDF]

Supplementary Table S1. The *Aspergillus nidulans* strains used in this study.

Strains	Genotype	Source
A4	wild-type, *veA+*	FGSC[a]
A1149	*pyrG89*; *pyroA4*; *nkuA*::*argB*	FGSC[a]
A1149 *pyrG*$^{+}$	*pyrG89*; *pyroA4*; *nkuA*::*argB*; *pyrG89*::*pyrG*^{Af}	This study
Δ*AN4405*	*pyrG89*; *pyroA4*; *nkuA*::*argB*; Δ*AN4405*::*pyrG*^{Af}	This study
Δ*AN4592*	*pyrG89*; *pyroA4*; *nkuA*::*argB*; Δ*AN4592*::*pyrG*^{Af}	This study[b]
Δ*AN5688*	*pyrG89*; *pyroA4*; *nkuA*::*argB*; Δ*AN5688*::*pyrG*^{Af}	This study[b]
Δ*AN7375*	*pyrG89*; *pyroA4*; *nkuA*::*argB*; Δ*AN7375*::*pyrG*^{Af}	This study[b]
Δ*AN8806*	*pyrG89*; *pyroA4*; *nkuA*::*argB*; Δ*AN8806*::*pyrG*^{Af}	This study[b]

[a]Fungal Genetics Stock Center; [b]already described in (Fernandes *et al*., 2016), but mutants were generated in-house.

Supplementary Table S2. List of primers used in *q*RT-PCR analyses.

Gene	Sequence 5´ – 3´	Sequence 5´ – 3´
lcbA	TAGTCCGGTGGTACGAGCA	AGTCACTCATGTCACCATACGA
AN1102	GGAGTTGGTCGCCAGCTTTG	AACCGGATGGAGGCATGGTT
AN1165	GACATCCGTCCAGACACTCA	AGTAAAGATGAGGTGTCGTCGA
basA	CCTCTACGTTCCATACGCCTA	TAGTGGAGAAGGTGAAGAACCA
barA	GTGGTGCTCAACCTGATGGA	TGGGTACATGATATGGCGTGA
lagA	TCCCCACAGAGAGCACGAA	GATGATGTGGTGGCCCACA
AN4405	CCTCGCTTTCCGGTCACCTA	CCGAGGGATTTGTGGTGGGT
AN4592	CGATGACCGCGTTCTCAAGG	GCCGAGCTTCTACGGAGTGT
AN5688	AGACACGACTGGGCCAACTT	GTGGTCACGGACCTGTTCCT
AN7375	GGACGCTTCGACTCCACTGT	TGCCTCAACGTTACCAACCCA
AN8806	TGACGACTGTTTGTGCTTTGGG	CCGGTCGAATAACAGTGACGTG
chsA	TGCAGTACGGACGTATATGG	CAGATACAGAACTGCATACGC
chsB	CTTGAACGTTTACGCCTTCAGC	TCGTCCAGACTCTTCTCTTCC
chsC	GCGATGTGGTTTTTGGTTGTGC	CATTGCGATATGCTGAACCTGC
chsD	ATGGAGCTGGTCTTGGTTCG	CCAAGAATAAGGGCGAGCAAG
chsF	TGGCTCACTACCTCGGTCATC	ACTTCATTTCCAAACGGCCAAC
chsG	GCCGTCACTGGATTCGACAA	CTGGACTCGTTCGGTGGACA
csmA	CCGACGAAGGAAAATTCGACC	GAGACATCCGAGACATATGCC
csmB	ACAGATAACCTTCTTCGACC	CGTCGTCTGAAGTCGTTGTTC
agsA	GCTTTCCAAATCCCACAGTTGG	GTGAAGCAGATATGCATCCGTG
agsB	ATCGGACACTACCTTCCCTG	GACTTGGCTGACGATCAACG
fksA	ATCTACTCGCTTAAGCAGTCCA	GCCACTCATTTTCAGGTTCAGA
actA	CTGGAAAGCGGTGGTATCCA	TGCATACGGTCGGAGATACC

Supplementary Table S3. Primers used in generation of gene-replacement mutants in *Aspergillus nidulans*.

Gene	Primer	Sequence 5´ – 3´
AN6157	AN6157_P1	AGCGATCATTAGTTATCCGGC
(pyrG)	AN6157_P2	TGAACGTCAACTTCGGAACAC
	AN6157_P3	ATCCACTTAACGTTACTGAAATCGGCGGTTCTCCAATGATTG
	AN6157_P4	GCTCCTTCAATATCATCTTCTGTCTGTGAGTGGAAATGTGTAACGG
	AN6157_P5	CAATGGAGAGGTGACCACTAGAG
	AN6157_P6	ACCGATGCGATTGTCAAGTG
AN4405	AN4405_P1	AGGAGGCTGGAATTGATGTC
	AN4405_P2	GCCGCTATCCAAGAAACTATG
	AN4405_P3	ATCCACTTAACGTTACTGAAATCCGAGGATAAAGGAGGAGAAGG
	AN4405_P4	GCTCCTTCAATATCATCTTCTGTCCGATTCGGATGATAACTGGG
	AN4405_P5	ACCAGGGTTTGCGACAGAG
	AN4405_P6	AAGCAAACCGACTACGAGACTC
AN4592	AN4952_P1	ACAATCAGCAGTCTCGGTAAGC
	AN4952_P2	ATTATCTTGGGCTGGGTTCTG
	AN4952_P3	ATCCACTTAACGTTACTGAAATCTGGCTGTCAAGCAATAACCAG
	AN4952_P4	GCTCCTTCAATATCATCTTCTGTCGTTATGCCATTGCCTTTGAC
	AN4952_P5	ATTCCGATGACGACGAACG
	AN4952_P6	GCCATCCTTTCTGCCCTC
AN5688	AN5688_P1	TTCGACGGTCCGTGCTC
	AN5688_P2	GGCAACGAGCGGACTATTC
	AN5688_P3	ATCCACTTAACGTTACTGAAATCGCAACGAATTGACCAAGACG
	AN5688_P4	GCTCCTTCAATATCATCTTCTGTCGTGCCTGAGTCGACAGATGAG
	AN5688_P5	GGCAGTCTCTTGAATTGACGAG
	AN5688_P6	CAGCAGGGTAGTAAGCGTGAC
AN7375	AN7375_P1	GCAGCCATGCTTCTCATACAG
	AN7375_P1	ATCCACTTAACGTTACTGAAATCAGTGAAGCCTGAAGGGATGG
	AN7375_P4	CTCCTTCAATATCATCTTCTGTCCTGCTGTTGGTGAATCTGTTATC
	AN7375_P5	CAAGACACCTGGCGTACCTAC
	AN7375_P6	TCTACCAACTGCTCGCCTTC
AN8806	AN8806_P1	TCAGGCACAGTCTCTGGAGG
	AN8806_P2	ATGCTTCAATCACTCATTCAGTC
	AN8806_P3	ATCCACTTAACGTTACTGAAATCGTGGTCAGGTCAGGTTCAGC
	AN8806_P4	CTCCTTCAATATCATCTTCTGTCGCACGGAGAAATAGAGCACG
	AN8806_P5	CCTCAGATCAATCCAAATCG
	AN8806_P6	CTATATCACGGCTGTGTTAGACG
$pyrG^{Af}$	CDS164	GATTTCAGTAACGTTAAGTGGAT
	CDS165	GACAGAAGATGATATTGAAGGAGC

Supplementary Table S4. PCR conditions used in generation of gene-replacement mutants.

PCR	Reaction mix	PCR conditions	Annealing Temp.
pyrG^{Af}	100 ng plasmid pCDS60<br>0.3 µM primers CDS164/CDS165<br>0.4 mM dNTPs NZYmix<br>1.25 U Proof DNA polymerase<br>50 µl final volume	denaturation: 95 ºC, 2 min<br>30 cycles:<br>denaturation: 95 ºC, 20 s<br>ramp to 70 ºC max. rate, 70 ºC to T*a* 0.1ºC/s<br>annealing: T*a*, 30 s<br>ramp to extension temperature: 0.2 ºC/s<br>extension: 68 ºC ,1 min/kb<br>final extension: 68 ºC, 5 min	*pyrg*^{Af}: 49 ºC
Flanking	100 ng *A. nidulans* A4 gDNA<br>0.3 µM primers P1/P3 or P4/P5<br>0.4 mM dNTPs NZYmix<br>1.25 U Proof DNA polymerase<br>50 µl final volume	denaturation: 95 ºC, 2 min<br>30 cycles:<br>denaturation: 95 ºC, 20 s<br>ramp to 70 ºC max. rate, 70 ºC to T*a* 0.1ºC/s<br>annealing: T*a*, 30 s<br>ramp to extension temperature: 0.2 ºC/s<br>extension: 72 ºC, 1 min/kb<br>final extension: 72 ºC, 5 min	*AN4405*: 51/53 ºC<br>*AN4592*: 53/51 ºC<br>*AN5688*: 52/51 ºC<br>*AN7375*: 53/51 ºC<br>*AN8806*: 52/52 ºC<br>*AN6157*: 51/53 ºC
Fusion	150 ng *pyrG*^{Af} cassette<br>150 ng 5'-flank<br>150 ng 3'- flank<br>0.5 µM primers P2/P5<br>0.5 mM dNTPs NZYmix<br>5.0 U Long DNA polymerase<br>50 µl final volume	denaturation: 95 ºC, 2 min<br>10 cycles:<br>denaturation: 95 ºC, 20 s<br>ramp to 70 ºC max. rate, 70 ºC to T*a* 0.1ºC/s<br>annealing: T*a*, 30 s<br>ramp to extension temperature: 0.2 ºC/s<br>extension: 68 ºC, 1 min/kb<br>5 cycles:<br>denaturation: 95 ºC, 20 s<br>ramp to 70 ºC max. rate, 70 ºC to T*a* 0.1ºC/s<br>annealing: T*a*, 30 s<br>ramp to extension temperature: 0.2 ºC/s<br>extension: 68 ºC, 1 min/kb, +5s/cycle<br>10 cycles:<br>denaturation: 95 ºC, 20 s<br>ramp to 70 ºC max. rate, 70 ºC to T*a* 0.1ºC/s<br>annealing: T*a*, 30 s<br>ramp to extension temperature: 0.2 ºC/s<br>extension: 68 ºC ,1 min/kb, +20s/cycle<br>final extension: 68 ºC, 10 min	*AN4405*: 51 ºC<br>*AN4592*: 52 ºC<br>*AN5688*: 51 ºC<br>*AN7375*: 53 ºC<br>*AN8806*: 50 ºC<br>*AN6157*: 52 ºC
Diagnostic	2 µl transformant gDNA<br>0.5 µM primers P2/P5<br>0.5 mM dNTPs NZYmix<br>5.0 U Long DNA polymerase<br>50 µl final volume	denaturation: 95 ºC, 2 min<br>10 cycles:<br>denaturation: 95 ºC, 20 s,<br>ramp to 70 ºC max. rate, 70 ºC to T*a* 0.1ºC/s<br>annealing: T*a*, 30 s<br>ramp to extension temperature: 0.2 ºC/s<br>extension: 68 ºC, 1 min/kb<br>5 cycles:<br>denaturation: 95 ºC, 20 s<br>ramp to 70 ºC max. rate, 70 ºC to T*a* 0.1ºC/s<br>annealing: T*a*, 30 s<br>ramp to extension temperature: 0.2 ºC/s<br>extension: 68 ºC, 1 min/kb + 5s/cycle<br>10 cycles:<br>denaturation: 95 ºC, 20 s<br>ramp to 70 ºC max. rate, 70 ºC to T*a* 0.1ºC/s<br>annealing: T*a*, 30 s<br>ramp to extension temperature: 0.2 ºC/s<br>extension: 68 ºC ,1 min/kb, + 20s/cycle<br>final extension: 68 ºC,10 min	*AN4405*: 51 ºC<br>*AN4592*: 53 ºC<br>*AN5688*: 52 ºC<br>*AN7375*: 53 ºC<br>*AN8806*: 52 ºC<br>*AN6157*: 52 ºC

Supplementary Table S5. Metabolic activity of *Aspergillus nidulans* grown for 24 hours and then exposed to dodecyltributylphosphonium chloride ([$P_{4\,4\,4\,12}$]Cl), 1-decyl-3-methylimidazolium chloride ([C_{10}mim]Cl) or cholinium decanoate, at the minimal inhibitory concentration, for 4 hours. Values represent the relative metabolic activity compared to the control (considered as 100%), followed by their standard deviation.

Ionic Liquid	Tested Concentration (mM)	Metabolic activity (%)
[$P_{4\,4\,4\,12}$]Cl	0.015	62.31 ± 3.07
[C_{10}mim]Cl	0.24	64.69 ± 2.82
Cholinium decanoate	3.0	96.72 ± 2.71

Supplementary Table S6. BLASTp analysis for serine palmitoyltransferase, 3-ketosphinganine reductase and dihydroceramide Δ4 desaturase functions in *Aspergillus nidulans*.

**Function**	**Serine palmitoyltransferase**	**3-ketosphinganine reductase**	**Dihydroceramide Δ4 desaturase**
Source organism	*Saccharomyces cerevisiae*	*Saccharomyces cerevisiae*	*Schizosaccharomyces pombe*
Encoding gene	*LCB2*	*TSC10*	*dsd1*
Source	Nagiec et al., 1994	Beeler et al., 1998	Garton et al., 2003
A. nidulans best hit	AN1102	AN1165	AN4405
e-value	0.0	$3.0e^{-27}$	$6.0e^{-4}$
Query coverage	96 %	90 %	93.0 %
Identity	54.8 %	30.4 %	54.0 %
Location (chromosome)	VIII	VIII	III
Gene model size	2019 bp	1224 bp	1381 bp
ORF size	2019 bp	1110 bp	1257 bp
Predicted protein size	672 aa	369 aa	418 aa

Supplementary Table S7. Gene expression analysis (*q*RT-PCR) of genes involved in the synthesis of sphingolipids, chitin, α-glucans and β-glucans after exposure to dodecyltributylphosphonium chloride ([$P_{4\,4\,4\,12}$]Cl), 1-decyl-3-methylimidazolium chloride ([C_{10}mim]Cl) or cholinium decanoate for 4 hours. Values represent the fold-change relative to time-zero followed by their standard deviation. Four biological replicates were performed. Values in bold are statistically significant compared to the control.

Gene	Control	[$P_{4\,4\,4\,12}$]Cl	[C_{10}mim]Cl	Cholinium decanoate
lcbA	1.017 ± 0.112	**1.717 ± 0.150*****	**1.783 ± 0.193*****	**2.004 ± 0.233*****
AN1102	1.046 ± 0.257	**4.988 ± 0.561*****	**2.117 ± 0.266****	**1.530 ± 0.260***
AN1165	0.890 ± 0.035	**1.412 ± 0.184****	0.885 ± 0.161	**1.936 ± 0.394****
basA	0.892 ± 0.184	**1.833 ± 0.307****	**1.320 ± 0.060****	**1.547 ± 0.458***
barA	0.901 ± 0.148	1.145 ± 0.205	0.853 ± 0.277	1.244 ± 0.393
lagA	1.108 ± 0.140	1.030 ± 0.292	0.883 ± 0.134	0.883 ± 0.219
AN4405	0.987 ± 0.108	**2.303 ± 0.542****	0.781 ± 0.129	1.221 ± 0.435
AN4592	0.801 ± 0.137	1.039 ± 0.174	**0.437 ± 0.071****	0.962 ± 0.206
AN5688	0.867 ± 0.048	1.074 ± 0.163	**0.422 ± 0.164****	0.789 ± 0.266
AN7375	0.929 ± 0.052	**2.041 ± 0.163*****	0.960 ± 0.052	1.182 ± 0.241
AN8806	1.036 ± 0.195	**4.789 ± 1.161*****	**2.050 ± 0.444****	**2.645 ± 0.631****
chsA	0.969 ± 0.051	**1.369 ± 0.177****	0.763 ± 0.198	1.026 ± 0.245
chsB	1.144 ± 0.063	**3.012 ± 0.227*****	1.712 ± 0.475	1.725 ± 0.955
chsC	0.897 ± 0.172	**0.463 ± 0.064****	**0.479 ± 0.124****	1.181 ± 0.331
chsD	1.077 ± 0.093	**3.222 ± 0.346*****	**1.601 ± 0.202****	**1.326 ± 0.138***
chsF	1.037 ± 0.310	**0.555 ± 0.182****	0.856 ± 0.149	0.949 ± 0.560
chsG	1.015 ± 0.187	1.109 ± 0.223	1.040 ± 0.163	0.891 ± 0.515
csmA	0.918 ± 0.077	**3.110 ± 0.111*****	1.156 ± 0.413	1.717 ± 1.043
csmB	0.983 ± 0.078	**1.240 ± 0.157****	1.042 ± 0.315	1.212 ± 0.307
agsA	0.856 ± 0.163	**2.043 ± 0.314*****	0.911 ± 0.308	1.126 ± 0.584
agsB	0.914 ± 0.160	**1.326 ± 0.161****	1.029 ± 0.237	1.292 ± 0.299
fksA	1.059 ± 0.094	**6.579 ± 0.372*****	**1.559 ± 0.125*****	**1.650 ± 0.226****

*: p-value < 0.05, **: p-value < 0.01, ***: p-value < 0.001.

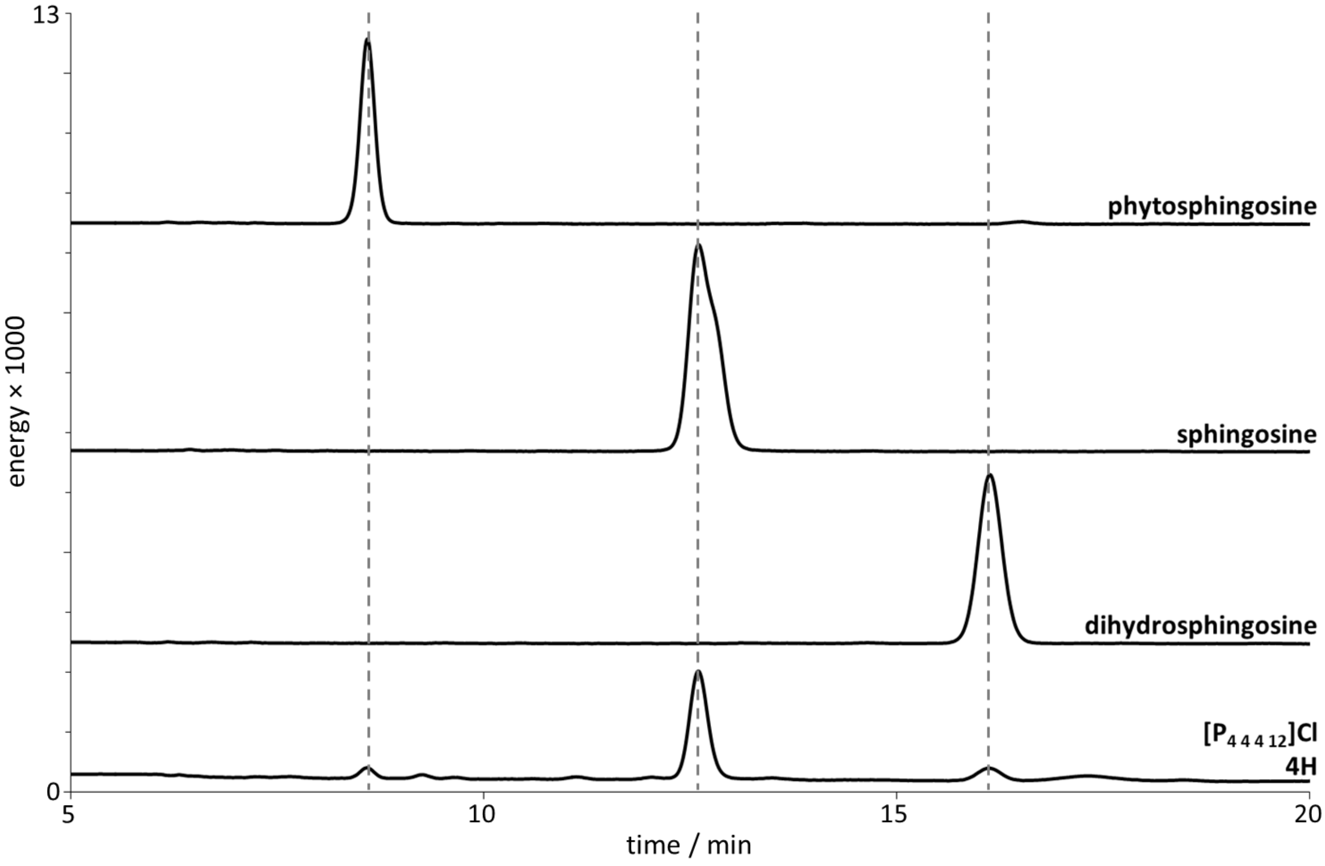

Supplementary Figure S1. Chromatographic profile (HPLC) of the standards phytosphingosine, sphingosine and dihydrosphingosine in comparison with an example of *A. nidulans* A4 sphingoid base profile after exposure to dodecyltributylphosphonium chloride ($[P_{4\,4\,4\,12}]Cl$), highlighting that the main sphingoid base accumulated corresponds to sphingosine.

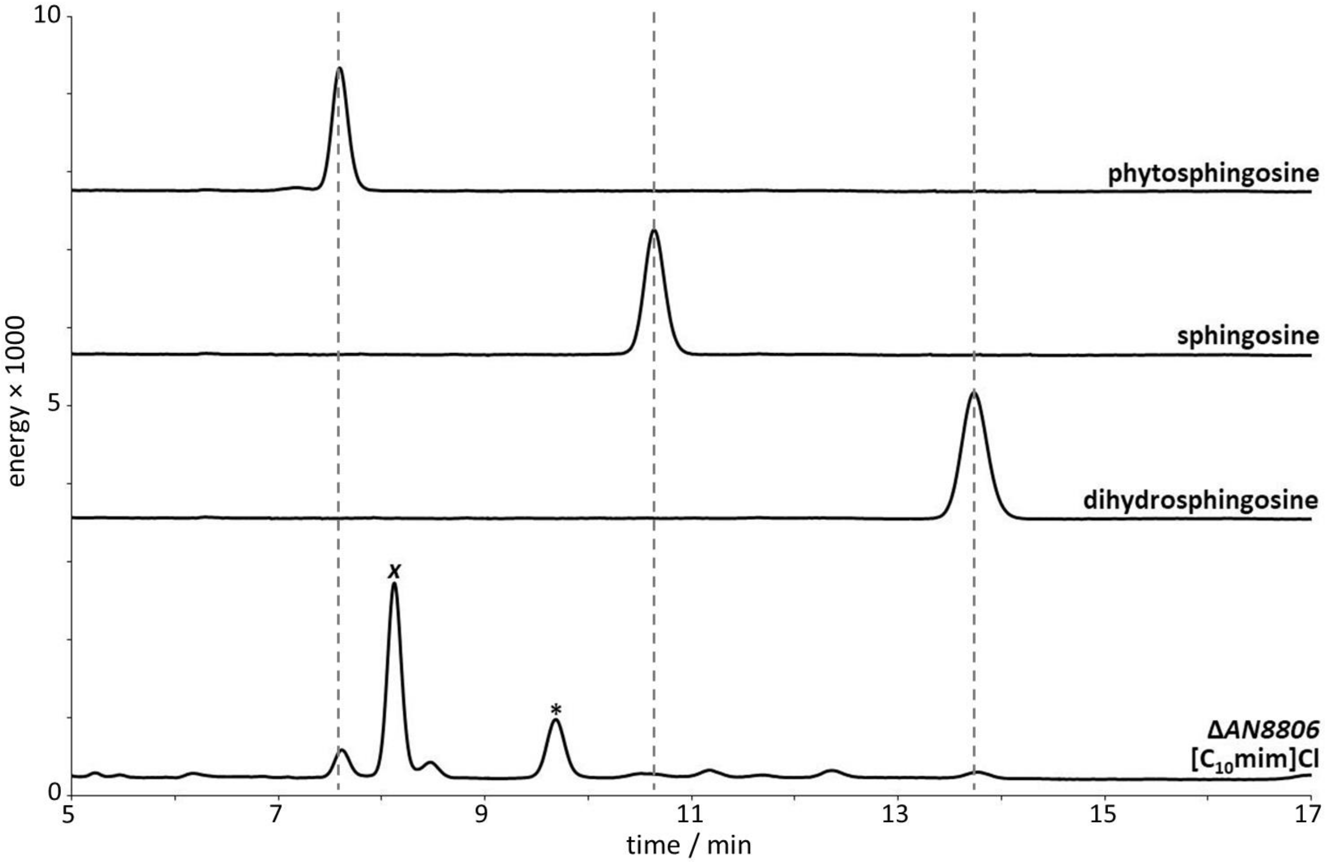

Supplementary Figure S2. Chromatographic profile (HPLC) of the standards phytosphingosine, sphingosine and dihydrosphingosine in comparison with an example of *A. nidulans* Δ*AN8806* sphingoid base profile after exposure to 1-decyl-3-methylimidazolium chloride ([C_{10}mim]Cl), highlighting that the main sphingoid base accumulated (sphingoid base *x*) does not corresponds to any of the tested standards. Only strain Δ*AN8806* accumulates sphingoid base indicated by *, which most likely corresponds to 9-methyl-4,8-sphingadiene, the main substrate of the ceramide glucosyltransferase.

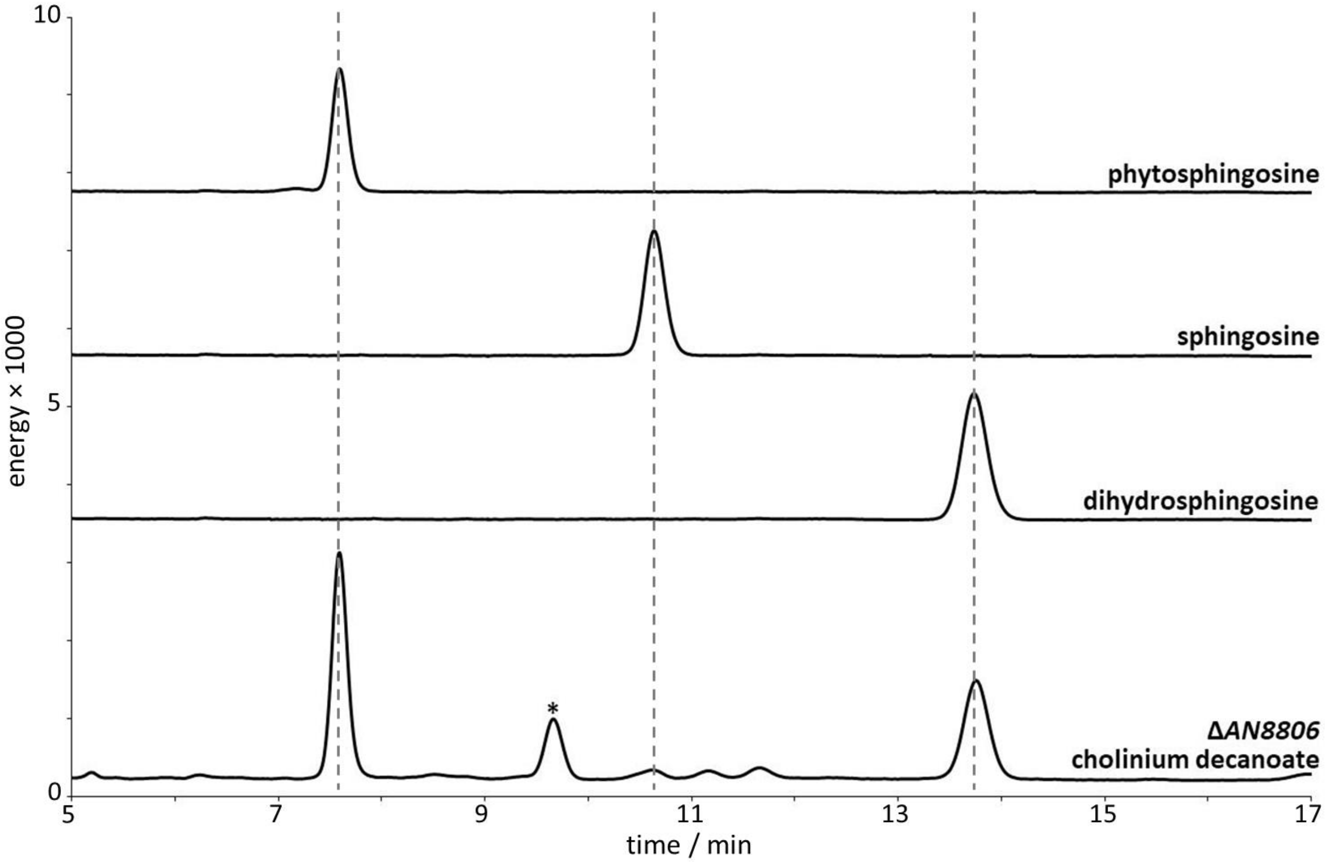

Supplementary Figure S3. Chromatographic profile (HPLC) of the standards phytosphingosine, sphingosine and dihydrosphingosine in comparison with an example of *A. nidulans* Δ*AN8806* sphingoid base profile after exposure to cholinium decanoate. Only strain Δ*AN8806* accumulates sphingoid base indicated by *, which most likely corresponds to 9-methyl-4,8-sphingadiene, the main substrate of the ceramide glucosyltransferase.

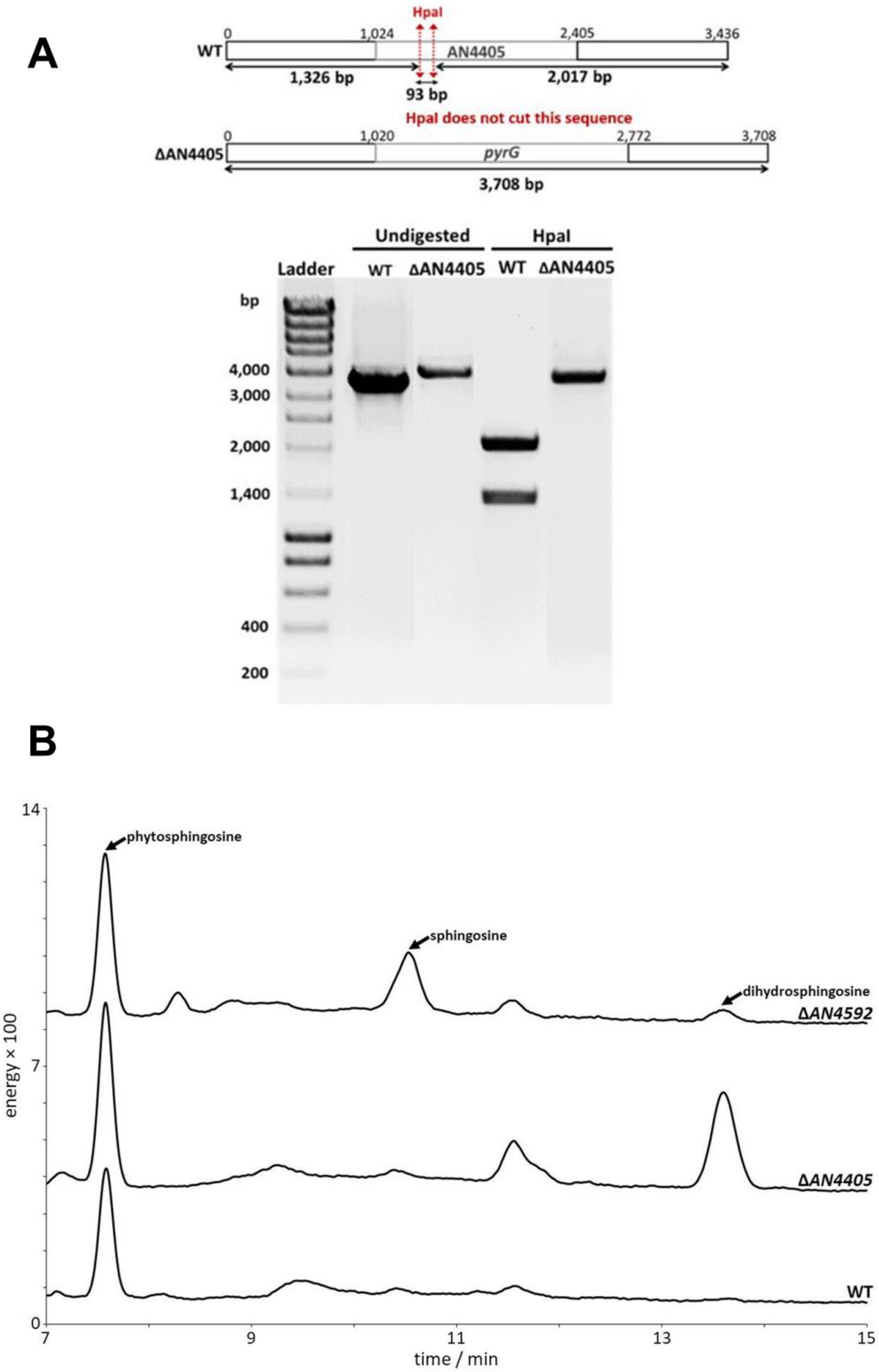

Supplementary Figure S4. Confirmation of generation of the Δ*AN4405* mutant. **(A)** Scheme of the amplified PCR products of wild-type and Δ*AN4405* transformant with their respective digestion sites with HpaI (red arrows, top) and agarose gel of digested and undigested PCR products (bottom). **(B)** Sphingoid base accumulation profile (HPLC) of mutants Δ*AN4405* and Δ*AN4592* grown for 24 hours without ionic liquids, compared to the wild-type strain. Strain Δ*AN4405* is impaired in the synthesis of sphingosine and accumulates the precursor sphingoid base, dihydrosphingosine.

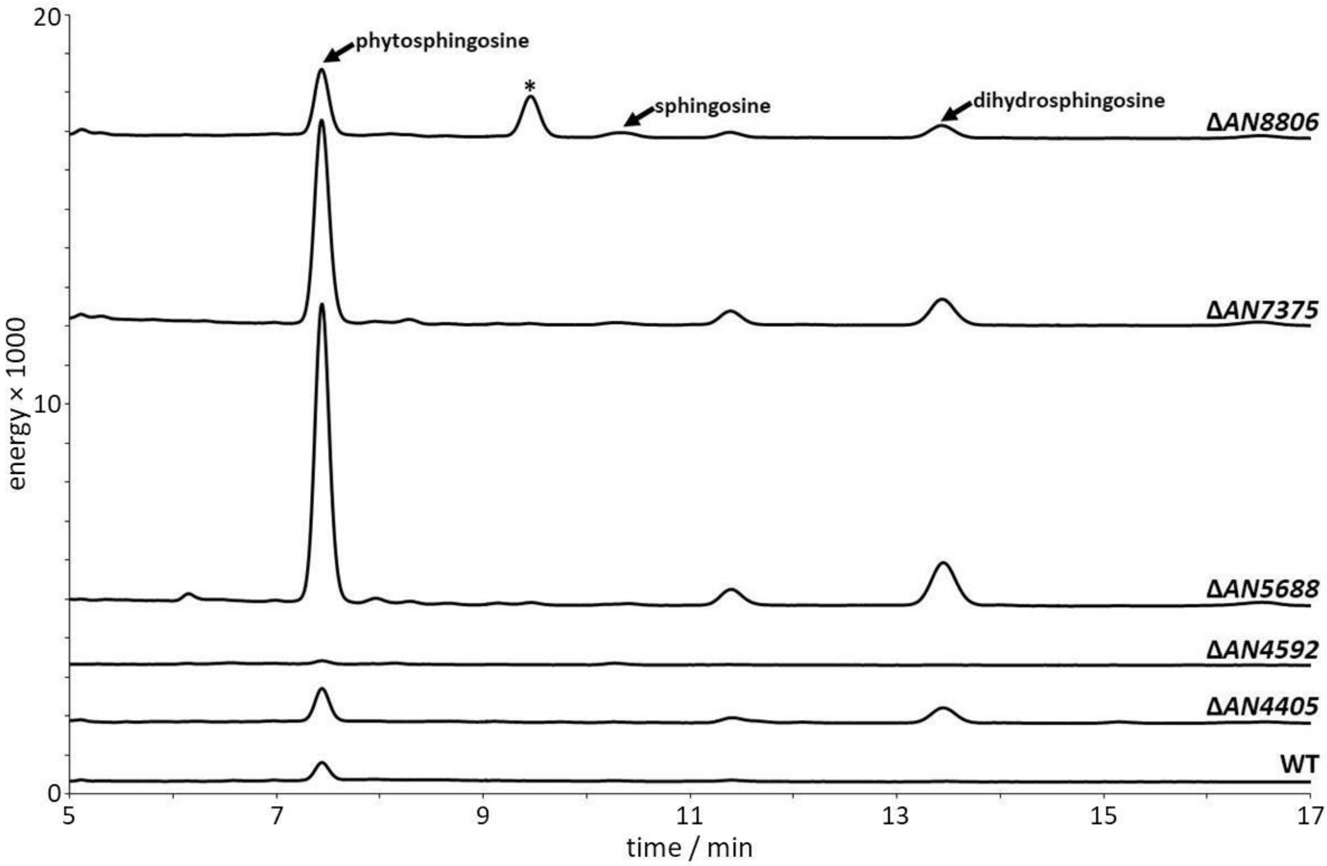

Supplementary Figure S5. Sphingoid base accumulation profile (HPLC) of deletion mutants of the glucosylceramide pathway grown for 24 hours without ionic liquid stress imposition, compared to the parental strain (wt). Strains ΔAN*5688* and Δ*AN7375* show the highest accumulation of phytosphingosine. Only strain Δ*AN8806* accumulates sphingoid base indicated by *, which most likely corresponds to 9-methyl-4,8-sphingadiene, the main substrate of the ceramide glucosyltransferase.
